# Closed-loop stimulation of lateral cervical spinal cord in upper-limb amputees to enable sensory discrimination: a case study

**DOI:** 10.1038/s41598-022-21264-7

**Published:** 2022-10-11

**Authors:** Ameya C. Nanivadekar, Santosh Chandrasekaran, Eric R. Helm, Michael L. Boninger, Jennifer L. Collinger, Robert A. Gaunt, Lee E. Fisher

**Affiliations:** 1 Rehab Neural Engineering Labs, University of Pittsburgh, 3520 Fifth Avenue, Suite 300, Pittsburgh, PA 15213 USA; 2 Department of Bioengineering, University of Pittsburgh, Pittsburgh, PA 15213 USA; 3 Center for Neural Basis of Cognition, Pittsburgh, PA 15213 USA; 4 Department of Physical Medicine and Rehabilitation, University of Pittsburgh, Pittsburgh, PA 15213 USA; 5 University of Pittsburgh Clinical Translational Science Institute, Pittsburgh, PA 15213 USA; 6 Human Engineering Research Labs, Department of Veteran Affairs, VA Center of Excellence, Pittsburgh, PA 15206 USA; 7 Department of Biomedical Engineering, Carnegie Mellon University, Pittsburgh, PA USA

**Keywords:** Biomedical engineering, Somatosensory system, Translational research

## Abstract

Modern myoelectric prosthetic hands have multiple independently controllable degrees of freedom, but require constant visual attention to use effectively. Somatosensory feedback provides information not available through vision alone and is essential for fine motor control of our limbs. Similarly, stimulation of the nervous system can potentially provide artificial somatosensory feedback to reduce the reliance on visual cues to efficiently operate prosthetic devices. We have shown previously that epidural stimulation of the lateral cervical spinal cord can evoke tactile sensations perceived as emanating from the missing arm and hand in people with upper-limb amputation. In this case study, two subjects with upper-limb amputation used this somatotopically-matched tactile feedback to discriminate object size and compliance while controlling a prosthetic hand. With less than 30 min of practice each day, both subjects were able to use artificial somatosensory feedback to perform a subset of the discrimination tasks at a success level well above chance. Subject 1 was consistently more adept at determining object size (74% accuracy; chance: 33%) while Subject 2 achieved a higher accuracy level in determining object compliance (60% accuracy; chance 33%). In each subject, discrimination of the other object property was only slightly above or at chance level suggesting that the task design and stimulation encoding scheme are important determinants of which object property could be reliably identified. Our observations suggest that changes in the intensity of artificial somatosensory feedback provided via spinal cord stimulation can be readily used to infer information about object properties with minimal training.

Limb loss has a profound impact on the ability of individuals to interact with their environment and perform activities of daily living. In addition to learning to use a prosthetic limb, people with upper-limb amputation have to devise ways of compensating for the lack of sensory feedback, even with state-of-the-art prosthetic devices. The current repertoire of prosthetics range from simple cosmetic hands to cable actuated hooks and dexterous robotic limbs. However, none of these devices provide tactile feedback, and as a result, intuitive control remains elusive^1,2^. In fact, users often prefer simpler body-powered prosthetics because they can infer information about limb state from pressure exerted on the residual limb^3^. Individuals with upper-limb amputation also rely on sustained visual attention to compensate for the lack of somatosensory feedback^4^. This reliance on visual cues leads to sub-optimal motor control in various situations such as attempting to grasp an object that is out of the line of sight, or rapidly modulating grip force to prevent object slipping^5^. Highlighting these challenges, surveys of upper-limb prosthesis users indicate restoring somatosensory feedback as a top unmet need^6–9^.

Normal haptic perception requires an interplay between tactile and proprioceptive modalities of sensory information^10^. Broadly, tactile sensation conveys information about object contact forces, temperature, and surface features of an object^10^. Meanwhile, proprioception conveys information about the state and orientation of the hand and fingers which enables stereognosis (i.e. inference of object location, shape, and size)^10^. Several studies have demonstrated the effectiveness of artificial somatosensory feedback in conveying these multiple modalities of information during prosthesis use. When tactile information was delivered by electrical stimulation of peripheral nerves in the residual limb, study participants demonstrated improvements in manipulating objects^11,12^, controlling grip force^13–15^, and identifying object compliance^16–19^. Further, electrical stimulation designed to mimic mechanoreceptor firing patterns enabled amputees to discriminate naturalistic textures^20,21^. More recently, feedback via peripheral nerve stimulation has been incorporated into a take-home system to demonstrate that subjects could learn to use this artificial somatosensory feedback in tasks of daily living^22–24^.

While tactile sensations are routinely evoked with electrical stimulation, reliable proprioceptive sensations have remained elusive. As a result, proprioceptive information has been conveyed by remapping the intensity of an evoked tactile sensation to a signal such as grasp aperture or finger joint angle. With these techniques, participants could discriminate object size with a success rate better than chance^17–19^.

Stimulation of the lateral cervical spinal cord can evoke focal sensations in the missing fingers and hand, even in people with high level amputations, such as at the proximal arm or shoulder^25^. In addition to conveying information about location, increasing the amplitude of spinal cord stimulation (SCS) led to linearly-modulated increases in percept intensity^25^. The primary goal of this case study was to determine if artificial somatosensory feedback provided by SCS could provide functionally relevant information during control of a prosthetic limb. Two subjects with upper-limb amputation interacted with objects of varying size and compliance using a sensorized DEKA hand^26,27^ or a virtual representation of that hand, rendered in MuJoCo^28,29^. Somatotopically-matched tactile sensory feedback was provided via lateral SCS by varying the stimulus amplitude in real-time. Subjects were asked to determine the size or compliance of the object based on this feedback. For each subject, the utility of feedback via SCS was inferred from the performance on the object discrimination task. Additionally, we characterized features of the closed-loop control system and task design that affected the utility of this feedback and performance on the discrimination task.

## Methods

### Study design

The aim of this case study was to use SCS to provide real-time sensory feedback so that two subjects could use a sensorized prosthetic hand to interact with objects and determine their size and compliance. To characterize the factors affecting the utility of sensory feedback, subjects performed an object discrimination task in two different control environments (real and virtual reality).

Subject 1 had a trans-humeral amputation of the right arm. Subject 2 had a trans-radial amputation of the right arm, and also had a right hemisphere stroke that resulted in extensive paralysis of the contralateral limb. The time since amputation was greater than two years for Subject 1 and three years for Subject 2. All procedures and experiments were approved by the University of Pittsburgh and Army Research Labs Institutional Review Boards and performed in accordance with relevant guidelines/regulations. Subjects provided informed consent before participation.

### Electrode implantation

SCS leads were implanted through a minimally invasive, outpatient procedure performed under local anesthesia, described previously^25^. Briefly, three 16-contact SCS leads (Infinion, Boston Scientific) were inserted percutaneously into the epidural space on the lateral aspect of the C5–C8 spinal cord. Contacts were 3 mm long, with 1 mm inter-contact spacing. Lead placement was iteratively adjusted based on the subjects’ verbal report of the location of sensations evoked by intraoperative stimulation. The leads were implanted for fewer than 29 days. Subjects attended testing sessions 3–4 days per week during the implantation period. For each subject, the majority of implant time was used to characterize perceptual characteristics of the evoked sensations^25^. The functional closed-loop experiments described here were performed during the last 5 days of the study.

### Neural stimulation

During testing sessions, stimulation was delivered using three 32-channel stimulators (Nano2+Stim; Ripple, LLC). The maximum current output for these stimulators was 1.5 mA per channel. To achieve the higher current amplitudes required for SCS, a custom-built circuit board was used to connect the output of groups of four channels together, thereby increasing the maximum possible output to 6 mA per channel. Custom software in MATLAB was used to trigger and control stimulation.

Stimulation pulse trains consisted of charge-balanced, anodic-first square pulses, with symmetric anodic and cathodic phases. Stimulation was performed either in a monopolar configuration, with the ground electrode placed at a distant location such as on the skin at the shoulder or hip, or in a multipolar configuration with one or more local SCS contacts acting as the return path. Stimulation frequencies and pulse widths ranged 1–300 Hz and 50–1000 µs, respectively. The interphase interval was 60 µs.

### Recording perceptual responses

The methodology for recording perceptual responses, characterizing their psychophysical properties, and determining their stability at threshold have been detailed elsewhere^25^. Briefly, after a one-second stimulation train, subjects used a touchscreen interface^30^ developed in Python to document the location and perceptual quality of the evoked sensation. The location of the sensory percept was recorded using a free-hand drawing indicating the outline of the evoked percept on an image of the appropriate body segment (i.e., hand, arm or torso). The percept quality was recorded using several descriptors that have been used previously to characterize evoked percepts^31,32^.

### Motor control of prosthetic hand

Subjects performed an object discrimination task using a sensorized DEKA hand (Mobius Bionics) or a virtual representation of that hand, rendered in MuJoCo^28,29^. Because neither subject was a regular prosthesis user and the time available for training was limited, each subject controlled the aperture of the prosthetic hand using a customized control signal. At the beginning of every day of testing, each subject was allowed to practice controlling the aperture of the prosthetic hand while interacting with objects and receiving artificial sensory feedback for up to 30 min. Subject 1 had a high trans-humeral amputation and did not have sufficient musculature in the residual limb to achieve reliable myoelectric control of the prosthesis. Instead, she wore a Data Glove (Fifth Dimension Technologies, 5DT) on her contralateral, intact hand, and the grasp aperture from the Data Glove was used to proportionally control the aperture of the real and virtual DEKA hand. Subject 2 had stroke-induced paralysis in her contralateral arm and could not use the Data Glove to control the DEKA hand. Because of this limitation and to more closely match the myoelectric approach that is commonly used clinically, bipolar surface EMG was recorded from the residual muscles in the ipsilateral forearm to control the prosthesis. EMG data were recorded at 2,000 Hz, high-pass filtered at 10 Hz, downsampled to 50 Hz by computing the moving average (20 ms bin size) and rectified. This rectified EMG signal was normalized to the peak EMG recorded during a maximum voluntary contraction prior to each session. Studies have shown that even in unilateral stroke, there is evidence of motor impairment in the ipsilateral limb; Subject 2 had difficulty achieving reliable prosthesis control, even with simple control schemes such as parallel dual-site control^33,34^. As such, we implemented a highly simplified control scheme, in which the subject was instructed to attempt a hand grasp, and when this signal crossed a manually defined threshold, the DEKA hand was commanded to close at a constant velocity of 15 degrees/s. Any time the processed EMG signal was below threshold, the hand was commanded to open at a constant velocity of 30 degrees/s. This behavior is similar to a normally-open body-powered prosthesis and a similar approach has been used previously by others to evaluate myoelectric robotic control^35,36^.

### Real-time somatosensory feedback via SCS

A subset of SCS electrodes that evoked focal percepts localized to the phantom hand and fingertips were used to provide real-time somatotopically-matched feedback during the object discrimination task. Sensors embedded in the fingers of the DEKA hand or virtual sensors in MuJoCo measured the force generated upon contact with the presented object. The maximum sensor force across the index, middle, and ring finger was mapped to an SCS electrode such that the receptive field of the evoked percept overlapped with the location of the sensors in the hand (Supplementary Fig. 1). Custom software was written in MATLAB (Mathworks, Natick, MA) to process the grasp force and control stimulation in real-time with an update rate of 50 Hz. Sensor signals were low-pass filtered using a 4th order Butterworth filter with a cutoff at 4 Hz. We implemented both a linear and an exponential stimulation encoding scheme between grasp force and stimulus amplitude. Subject 1 performed the object discrimination task using both stimulation encoding schemes. Her performance using each encoding scheme is reported separately. Subject 2 performed the object discrimination task using the linear encoding scheme only. For the linear encoding scheme, the grasp force was first normalized to a scale ranging from 0 to 1 as shown in Eq. (1),

1 $$F_{n} = \left( {F - F_{min} } \right)/\left( {F_{max} - F_{min} } \right)$$

where ${F}_{n}$ is the normalized grasp force, $F$ is the instantaneous grasp force, and ${F}_{max}$ and ${F}_{min}$ are the upper and lower limits of the grasp force measured by the sensor, respectively. The instantaneous stimulation amplitude ($A$) was determined as shown in Eq. (2):

2 $$A = F_{n} \times \left( {A_{max} - A_{min} } \right) + A_{min}$$

where ${A}_{max}$ and ${A}_{min}$ are the upper and lower limits of the stimulus amplitude.

For the exponential encoding scheme, the instantaneous stimulation amplitude ($A$) was determined as shown in Eq. (3):

3 $$A = \left\{ {\begin{array}{*{20}c} {A_{min} .e^{{{\upomega }F}} , F > 1} \\ {0 , F \le 1} \\ \end{array} } \right.$$

where $F$ is the instantaneous grasp force measured by the sensor, $\upomega$ is an empirically assigned scaling factor (0.005 to 0.025), and ${A}_{min}$ is the lower limit of the stimulus amplitude.

### Object discrimination task design

The overall goal of this case study was to provide functionally relevant somatosensory feedback. Due to the limited duration of these experiments, several parameters (such as the choice of stimulation encoding scheme, grasp force threshold, object geometry) were adjusted empirically across testing sessions and between subjects to improve the quality of the artificial somatosensory feedback. Table 1 provides a summary of the control scheme and number of object presentations for the physical and virtual object discrimination task for Subjects 1 and 2. Below, we provide more detail about the implementation and assessment of each of these tasks.

**Table 1:** Summary of performance on object discrimination task for each subject.

Subject	Control scheme	Control environment	Size discrimination		Compliance discrimination	
			Accuracy	# Object presentations	Accuracy	# Object presentations
1	Contralateral Data glove	virtual DEKA	74%	72	46%	90
		real DEKA	58%	55	–	–
2	Ipsilateral EMG	virtual DEKA	–	–	51%	75
		real DEKA	27%	30	60%	60

### Virtual DEKA hand in MuJoCo

**Figure 1:**
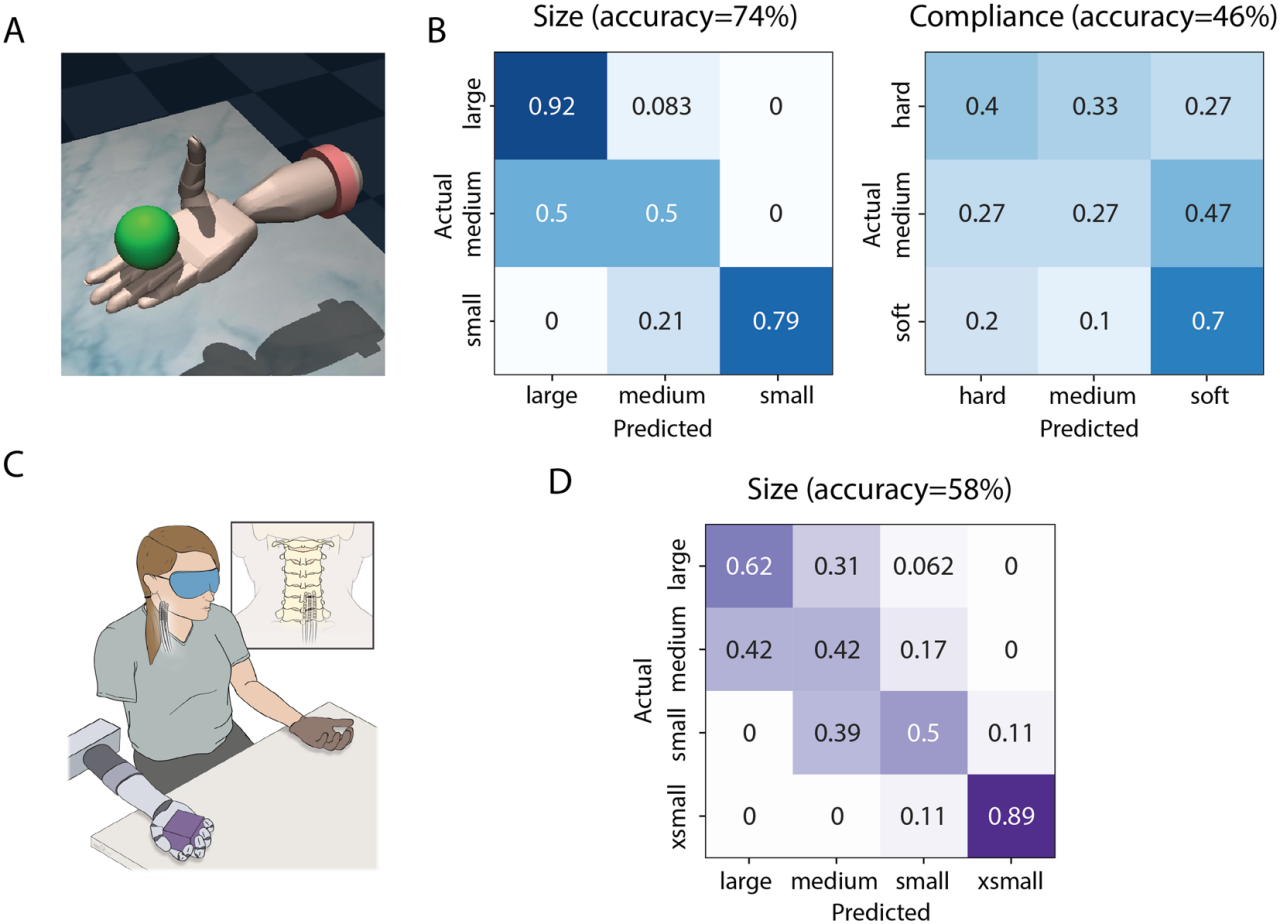
Object discrimination results for Subject 1. This subject used the DataGlove to control both virtual and physical prosthetic hands. (**A**) Representation of the DEKA hand in the MuJoCo virtual environment with a spherical object. (**B**) Confusion matrices for the object discrimination task using the virtual DEKA hand and an exponential stimulation encoding scheme (n = 72 for size, n = 90 for compliance). (**C**) Experimental setup for the object discrimination task with the physical DEKA hand and DataGlove (n = 55). (**D**) Confusion matrix for the object size discrimination task using a linear stimulation encoding scheme. The compliance discrimination task with the DEKA hand was not performed for this subject. Illustration in (**C**) created by Kenzie Green and published under a CC BY open access license.

Both subjects used a virtual representation of the DEKA hand to perform the object discrimination task in a virtual environment designed using MuJoCo^28,29^. Subject 1 was presented with 9 spheres of three different sizes (small, medium, and large) and compliances (soft, medium, and hard) (Fig. 1A). The subject had 10 s to interact with the object without visual feedback and an exponential stimulation encoding scheme between sensor force and stimulation was used for all object presentations. For the size discrimination task, each sphere was presented 8 times in random order resulting in a total of 72 object presentations. Both size and compliance were varied across trials, however, the subject was only asked to identify object size. Each size was presented 24 times. For the compliance discrimination task, each object was presented 10 times in random order resulting in a total of 90 object presentations. Both size and compliance were varied across trials, however, the subject was only asked to identify object compliance. Each compliance level was presented 30 times.

**Figure 2:**
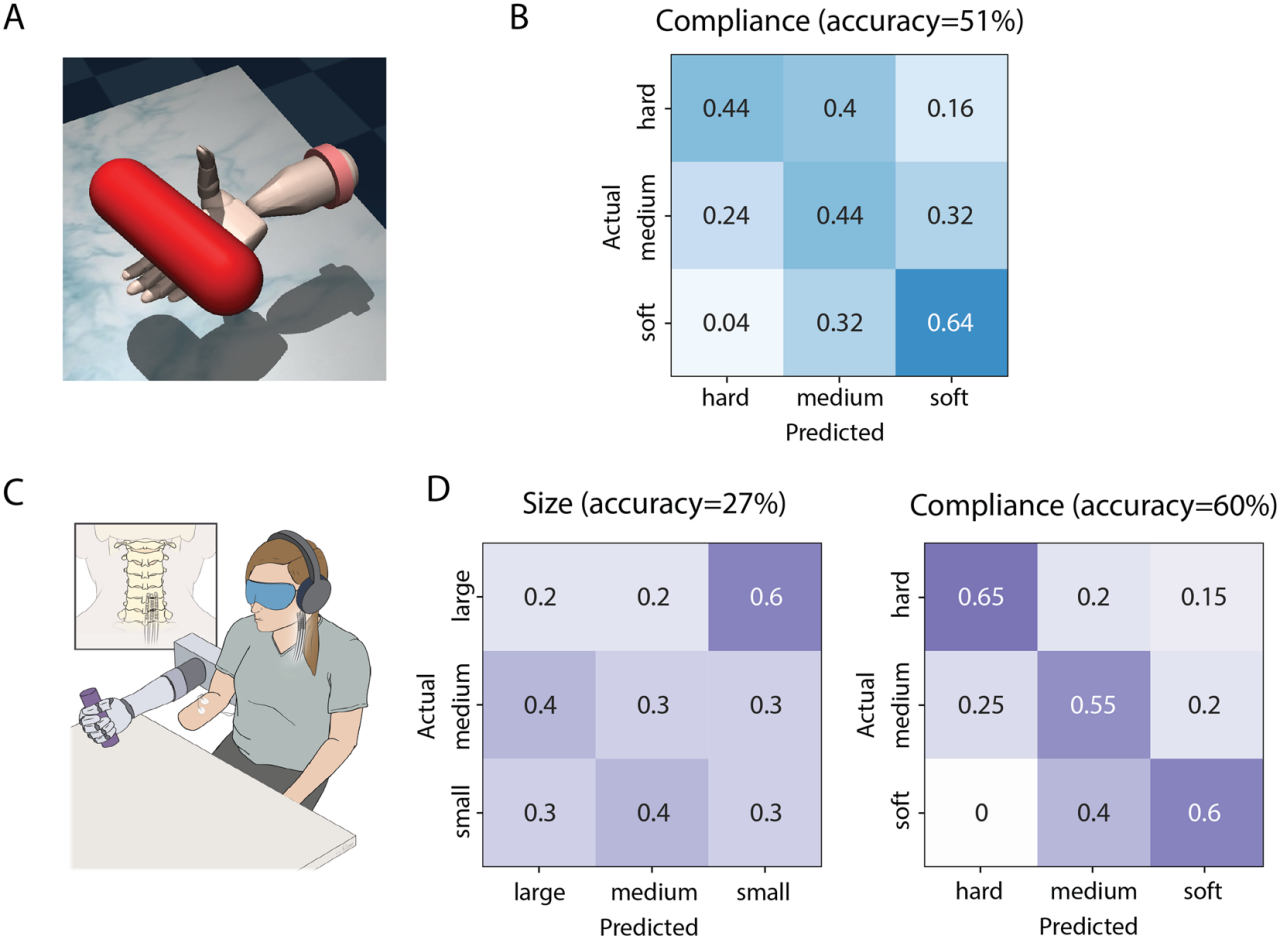
Object discrimination results for Subject 2. This subject used ipsilateral EMG signals to control closing of both the virtual and physical prosthetic hands. (**A**) Representation of the DEKA hand in the MuJoCo virtual environment with a cylindrical object. (**B**) Confusion matrix for the compliance discrimination task performance with the virtual DEKA hand using a linear stimulation encoding scheme (n = 75). The size discrimination task with the virtual DEKA hand was not performed for this subject. (**C**) Experimental setup for the object discrimination task with the physical DEKA hand and ipsilateral EMG electrodes. (**D**) Confusion matrices for the object discrimination task using a linear stimulation encoding scheme (n = 30 for size, n = 60 for compliance). Illustration in (**C**) created by Kenzie Green and published under a CC BY open access license.

For Subject 2, cylinders of three different sizes and compliance levels were presented (Fig. 2A). The subject had 10 s to explore the object without visual or auditory feedback. A linear encoding scheme between sensor force and stimulation was used for all object presentations. For the compliance discrimination task, three different compliances (all with large size) were presented 25 times in random order resulting in a total of 75 object presentations.

### Physical DEKA hand

For the physical DEKA hand, Subject 1 was presented with cubes of four different sizes (extra small, small, medium, and large), all made from the same foam rubber material (Fig. 1C). The presentation order was randomized, and the subject performed the task without visual feedback. A timeout for object exploration was not enforced, however the subject never explored an object for more than 10 s. A linear encoding scheme between sensor force and stimulation was used for 9, 18, 12, and 16 presentations of the four sizes, respectively.

Subject 2 was presented with cylinders of three different sizes (small, medium, and large) or compliances (soft, medium, and hard) (Fig. 2C). The design of these objects was changed from cubes to cylinders to reduce the slippage between the fingers and the corners of objects that we sometimes observed in Subject 1. The subject was given 20 s to explore each object. For the size discrimination task, objects of three different sizes (all with hard compliance) were presented 10 times in random order resulting in a total of 30 object presentations. For the compliance discrimination task, three different compliances (all with medium size) were presented 20 times in random order resulting in a total of 60 object presentations. The subject performed the task without visual feedback while wearing noise-cancelling headphones. A linear encoding scheme between sensor force and stimulation was used for all object presentations.

Supplementary Videos 1and 2 show an example of a single object presentation along with the corresponding grasp force readout and real-time stimulus modulation for each subject.

### Statistical analysis

To test whether the probability of success on the object discrimination task was above chance levels we used a binomial test (*scipy* package in Python) for each object size and compliance^37^. The null hypothesis for the binomial test was that accuracy for each task was no better than 33% for Subject 1 and 2 using the virtual DEKA, 33% for Subject 2 using the real DEKA hand and 25% for Subject 1 using the real DEKA hand.

To explore which features of the task were most correlated with successfully identifying object size or stiffness, we characterized the stimulation onset delay, peak stimulation amplitude, and the rate of change of stimulation for each object presentation. Additionally, for Subject 1 we characterized the contralateral hand aperture for each object presentation. For each subject, separate multivariate analyses of variance (MANOVA) were performed for object discrimination tasks involving the real and virtual DEKA hand. Size and compliance were the independent variables and hand aperture at stimulation onset, peak amplitude of stimulation and the rate of change of stimulation were the dependent variables. Subsequent univariate analysis (ANOVA) was carried out for each independent variable and *post-hoc* (Tukey-HSD) tests were carried out for dependent variables that displayed a significant effect. The Pearson correlation coefficient (*p*) between dependent variables that showed a significant difference across all objects was used to plot standard deviational ellipses. This allowed us to identify how object size and compliance were encoded through stimulation and postulate on the strategy employed by each subject during the object discrimination task. All statistical analyses were carried out using the *statsmodel* package in Python^37^.

## Results

### SCS evokes sensory percepts localized to the missing limb

We selected a subset of electrodes that evoked percepts in the phantom hand to provide somatotopically-matched sensory feedback in real-time as subjects interacted with objects of varying size and compliance. Supplementary Fig. 1C shows representative sensory percepts localized to the missing hand that were evoked by stimulation through these electrodes. A complete overview of the quality, stability, and psychophysical properties of percepts evoked via SCS has been detailed previously^25^.

### SCS provides functionally relevant somatosensory feedback

#### Subject 1: Object discrimination task performance

In the MuJoCo virtual environment (Fig. 1A), Subject 1 was most successful in determining the size of the objects, with an overall accuracy of 74% (*p* < 0.001, binomial test) (Fig. 1B) across all 72 object presentations. There was a slight decrease in accuracy following the first day of testing (day one: 72%, day two: 61%), although the highest overall accuracy within a single set of 5 random presentations of each object size was 94% and occurred on the last day of testing. Across multiple sessions, the subject correctly identified large and small objects (92% and 79% accuracy respectively; *p* < 0.001, binomial test) more often than medium objects (50%, *p* < 0.001, binomial test; chance level = 33%). Interestingly, when the subject misidentified medium-sized objects, they were always incorrectly identified as large-sized objects. When performance on the size discrimination task was analyzed for each object compliance (Supplementary Fig. 2A), Subject 1 had the highest accuracy when presented with soft objects (95%, *p* < 0.001, binomial test) as opposed to medium and hard objects (71%, *p* < 0.001 and 54%, *p* = 0.026, binomial test). Objects with stiffer compliance were frequently misidentified as larger sizes.

The subject was less accurate when determining object compliance with an overall accuracy of 46% (*p* = 0.015, binomial test), across all 90 object presentations. Across 4 days of testing, there was no change in the overall accuracy (day one: 44%, day 2: 44%, day 3: 50%, day 4: 44%). However, the subject was consistently more successful in identifying the soft object (70% accuracy, *p* < 0.001, binomial test; 36% false positive rate). Furthermore, when identifying object compliance for different object sizes (Supplementary Fig. 2B), the subject performed below chance levels when presented with small objects (overall accuracy 13%, *p* = 0.99, binomial test) but performance improved for large objects (67% accuracy, *p* < 0.001, binomial test). Interestingly, for medium and large objects the subject correctly identified soft objects with a high accuracy (100% and 80%) but she could only correctly identify all three compliances at above chance levels for the large object (70%, 50%, and 80% for soft, medium, and hard respectively).

The subject also used a physical DEKA hand to explore and identify objects of four different sizes with an overall accuracy of 58% (*p* < 0.001, binomial test) (Fig. 1C) across all 55 object presentations. Across 2 days of testing, there was no change in the overall accuracy (day one: 60%, day 2: 54%). The subject achieved accuracy rates of 62% and 89% with the largest and smallest objects (*p* = 0.002 and *p* < 0.001, binomial test; chance level = 25%), respectively. The confusion matrix (Fig. 1D) shows that, for the two intermediate sizes, the subject commonly misidentified them as objects of adjacent larger sizes. This trend was similar to the misidentification of intermediate-sized objects observed in the virtual environment.

#### Subject 2: Object discrimination task performance.

Subject 2 used the virtual DEKA hand to identify object compliance with an overall accuracy of 51% (*p* < 0.001, binomial test Fig. 2B). Across 2 days of testing, there was a slight increase in the overall accuracy (day one: 40%, day two: 57%). The highest overall accuracy within a single set of 5 random presentations of each object size was 60%. However, objects of adjacent compliance were frequently misidentified and only the soft objects were identified with an accuracy greater than 50% (*p* = 0.001, binomial test). Subject 2 also used the physical DEKA hand to identify object compliance with an overall accuracy of 60% (p < 0.001, binomial test). The confusion matrix in Fig. 2D shows that for this task, the subject was able to identify the soft, medium, and hard objects with accuracies of 60%, 55%, and 65% (*p* = 0.01, *p* = 0.03, *p* = 0.003, binomial test), respectively. Performance on the compliance discrimination task was similar for small (67%, *p* = 0.007, binomial test) and medium (60%, *p* = 0.002, binomial test) objects (Supplementary Fig. 3). While accuracy for large objects was similar to the other two objects, the result was not significantly different from chance (54%, *p* = 0.08, binomial test), possibly because there were fewer trials with large objects. There was no change in the overall accuracy across all days of testing.

Compared to compliance discrimination, the subject identified object size with a lower overall accuracy (27% *p* = 0.82, binomial test,) across 30 object presentations. The false positive rates for small, medium, and large objects were 20%, 40% and 50% respectively.

### Object size and compliance are encoded by independent stimulation features

We performed a post-hoc analysis of the sensor signals recorded during the object discrimination task to determine which features were most strongly correlated with subjects’ ability to accurately identify object compliance or size. Supplementary Fig. 4shows examples of the sensor signal and the instantaneous stimulation current profile during the object discrimination task for both subjects.

#### Subject 1

**Figure 3:**
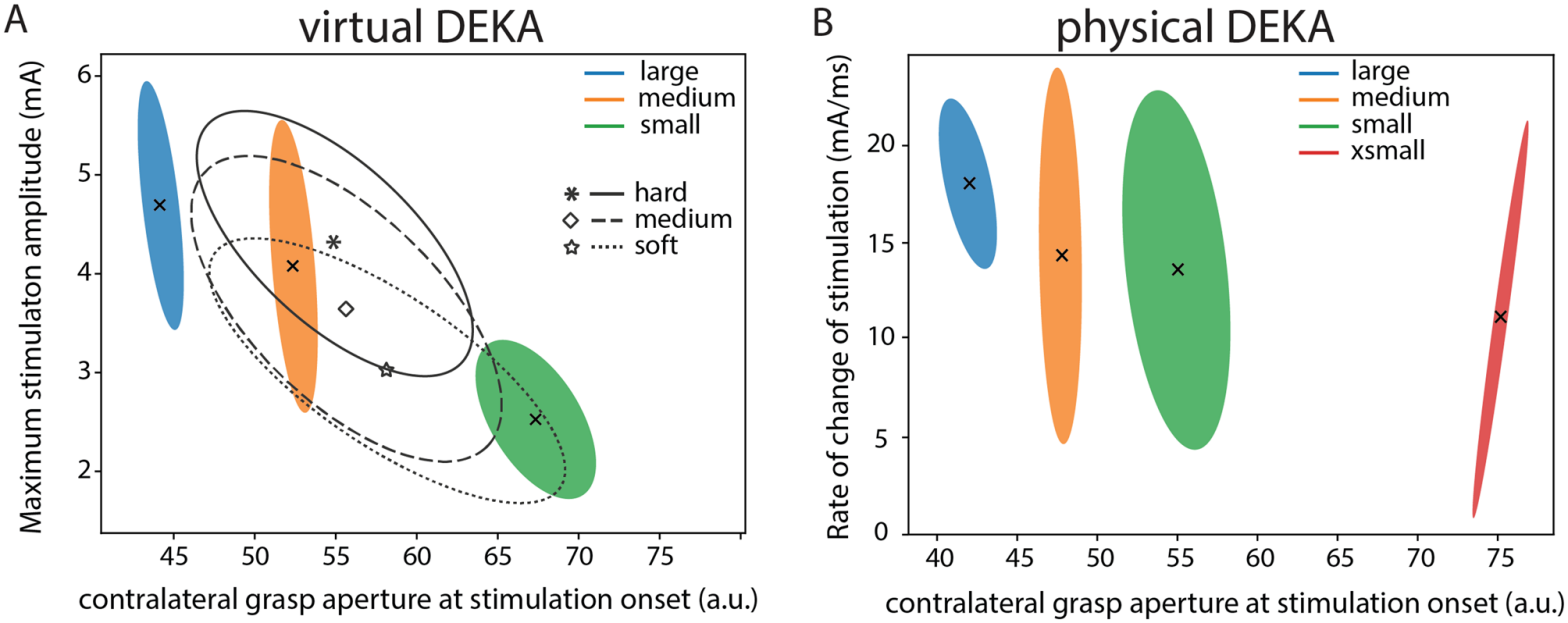
Salient features of stimulation correlate with subjects’ ability to discriminate object size or stiffness. Standard deviational ellipses for (**A**) the maximum stimulation amplitude and contralateral (DataGlove) grasp aperture at stimulation onset when using the virtual DEKA hand and (**B**) the rate of change of stimulation and contralateral grasp aperture at stimulation onset, when using the physical DEKA hand for Subject 1.

For Subject 1, when using the virtual DEKA hand, the contralateral hand aperture at stimulation onset was significantly different (*p* < 0.001, ANOVA) for each object size. There was no overlap in the standard deviational ellipses for grasp aperture for all object presentations (Fig. 3A). Subject 1 may have attended to the aperture of her intact contralateral hand at stimulation onset to reliably perform the size discrimination task. It is also possible that Subject 1 utilized the timing of object contact to determine object size. Analysis of the lag between the onset of the grasp command and the onset of stimulation revealed that the delay in stimulation onset was significantly different (*p* = 0.003, ANOVA) when comparing small objects to either large or medium objects only (Supplementary Fig. 5). Therefore, the timing of stimulation onset and contralateral grasp aperture both may have contributed to her performance on this task. The combination of multiple features may also explain the subject’s high overall accuracy on the size discrimination task (74% accuracy).

Further univariate analysis of object compliance revealed that the rate of change of stimulation was significantly different (*p* < 0.001, ANOVA) when comparing hard objects to either soft or medium objects while the peak stimulation amplitude was significantly different for hard and soft objects only (*p* = 0.003, ANOVA) (Supplementary Fig. 5). Subject 1 may have attended to these features of stimulation to identify hard and soft objects (70% and 40% respectively) with a greater accuracy than objects with medium compliance (27%).

With the physical DEKA hand for Subject 1, object size was the only independent variable since all objects had a medium compliance. Similar to observations with the virtual DEKA hand, the contralateral hand aperture at stimulation onset was significantly different (*p* < 0.001, ANOVA) for each object size and there was no overlap in the distribution of contralateral grasp apertures across all object presentations (Fig. 3B). Further analysis of the lag between the onset of the grasp command and the onset of stimulation revealed that the delay in stimulation onset was significantly different (*p* = 0.005, ANOVA) when comparing extra small objects to either large or medium objects only (Supplementary Fig. 5). This result indicates that Subject 1 could identify object size reliably from contralateral grasp aperture at stimulation onset alone.

#### Subject 2

**Figure 4:**
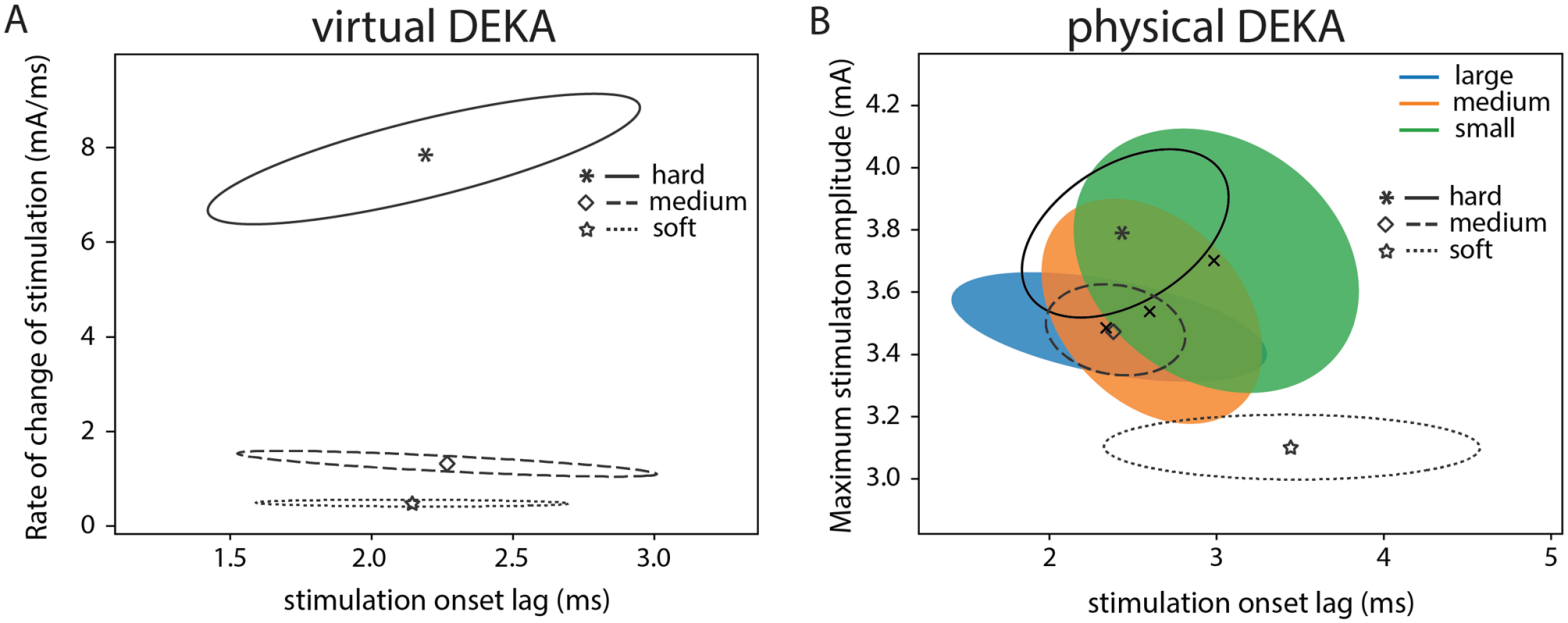
Salient features of stimulation correlate with subjects’ ability to discriminate object size or stiffness. Standard deviational ellipses for (**A**) the rate of change of stimulation and stimulation onset lag when using the virtual DEKA hand and (**B**) the maximum stimulation amplitude and stimulation onset lag, when using the physical DEKA hand for Subject 2. The color of the ellipses represent object size and the line style represents object compliance. The centroid of each standard deviational ellipse represents the mean of the distribution for each object size and compliance.

For Subject 2, when using the virtual DEKA hand, object compliance was the only independent variable since all objects were of medium size. A one-way ANOVA and subsequent post-hoc analysis confirmed that there was a significant difference in the rate of change of stimulation for each compliance (*p* < 0.001, ANOVA). For all object presentations there was no overlap in the standard deviational ellipses for the rate of change of stimulation (Fig. 4A). This result indicates that the subject may have relied on the rate of change of stimulation to determine object compliance. However, the overall low accuracy on the compliance detection task (51%) may be attributed to inherent difficulty in reliably detecting this stimulation feature.

For the object discrimination task with the physical DEKA hand, the rate of change of grasp force was similar for all objects since the hand was commanded to close at a constant velocity. Similar to observations in Subject 1, the aperture of the DEKA hand at stimulation onset was significantly different (*p* < 0.001, ANOVA) for each object size (Supplementary Fig. 6). However, there was no statistically significant relationship between grasp aperture and any feature of stimulation. For all object presentations, there was significant overlap in the standard deviational ellipses for the lag between the onset of the grasp command and the onset of stimulation (Fig. 4B). Object size could not be conveyed to the subject via this control scheme, which likely explains the subject’s decreased performance on the size discrimination task. However, univariate analysis showed that there was a statistically significant relationship between object compliance and the peak stimulation amplitude (*p* < 0.01, ANOVA). Post-hoc tests showed that the peak stimulation amplitude was significantly different for all object compliances (*p* < 0.001). Therefore, Subject 2 may have used stimulation amplitude to identify different object compliances.

## Discussion

### Subjects can use somatosensory feedback via SCS during an object discrimination task

In this case study of two subjects with upper-limb amputation, we demonstrate that somatotopically-matched real-time feedback provided by SCS can be used to determine object size or compliance. Subject 1 was consistently more adept at determining object size (up to 74% accuracy) while Subject 2 achieved a higher accuracy level in determining object compliance (up to 60% accuracy). Both subjects could readily use the sensory feedback with minimal training and their performance on the object discrimination task did not change across multiple days of testing. Several research groups have carried out similar experiments exploring the usability of artificial somatosensory feedback, especially as provided by peripheral nerve stimulation^15,16,18,19^. Some of these studies have shown comparable results but with more consistency across their subject population^18,19^. Other studies demonstrated that somatosensory feedback provided via transcutaneous electrical neural stimulation (TENS) can also be used to discriminate between objects. These studies have either chosen pre-programmed stimulation profiles^38^ or the stimulation parameters were modulated significantly enough to change the modality of the sensation^39^ while interacting with some of the objects. Similar to the results from our study, performance with TENS was limited to discriminating only one property of the object at a time.

The performance of our subjects varied based on the environment (real or virtual DEKA hand) and the control strategy (contralateral grasp aperture control or ipsilateral linear velocity myoelectric control).

Nevertheless, artificial somatosensory feedback delivered by SCS would be uniquely beneficial to people with more proximal amputations (i.e. transhumeral or shoulder disarticulation), wherein peripheral nerve stimulation would not be feasible. Further, we used commercially-available SCS electrodes that are already being implanted in many thousands of people each year, which may help to accelerate translation of this approach to the clinical. These SCS electrodes were not, however, designed for achieving highly focal nerve activation. Thus, we were limited in the number of electrodes evoking unique and relevant percepts that could be used to provide somatosensory information during the task. Moreover, both subjects’ lack of experience with upper-limb prosthetics and the need to explant the electrodes after 29 days significantly curtailed the time available for subjects to familiarize with the artificial feedback and the task. We believe that, in the future, new designs for SCS electrodes and the use of fully implanted systems will allow subjects to achieve far better results than those reported here.

### Utility of somatosensory feedback is dependent on task design

While both subjects were able to incorporate sensory feedback into their use of a prosthetic device, success rates for identifying object size and stiffness were starkly different between subjects.

Previous studies have shown that a signal that conveys grasp aperture information is key to determining object size when using a prosthesis^15,18,40^. Providing true proprioceptive information through artificial somatosensory feedback has consistently been a difficult challenge for somatosensory neuroprostheses^2^. Only a few studies have reported that electrical stimulation evoked true proprioceptive sensations that could be reliably modulated with specific stimulation parameters^41^. To overcome this obstacle, artificial proprioception information has been provided by either mapping prosthesis grasp aperture information to a tactile sensation^18,19^, using objects that were specifically designed to provide timing differences across multiple channels of sensory feedback^40^, or mapping grasp aperature to the intensity of the sensation of movement of a specific finger or joint^19^. In multiple of these studies, performance in object size discrimination decreased to chance levels when proprioception information was withdrawn^18,19^.

Subject 1 had proportional control of the DEKA hand through a DataGlove on her contralateral intact hand. This control strategy provided her with information about grasp aperture through the intact proprioceptive pathways in the contralateral limb. Combined with the timing of stimulation onset, this proprioceptive information provided a reliable estimate of object size. Additionally, Subject 1 did not wear noise-cancelling headphones when performing the task using the real DEKA hand. However, performance on the object discrimination task was comparable using the virtual and physical DEKA hand, suggesting the presence or lack of audio feedback did not affect the experiment.

Meanwhile, Subject 2 used an EMG signal to control the closing velocity of the prosthetic hand such that grasping the object required a sustained finger/wrist flexion. In the absence of proprioceptive feedback, Subject 2 may have attended to the time delay between the onset of wrist/finger flexion and the onset of stimulation to infer the grasp aperture at object contact. However, the subject had no feedback about when the signal exceeded the threshold, leading to variability between the onset of flexion and the onset of grasp. This variability resulted in objects of different sizes having overlapping stimulation onset lags and likely explains her decreased accuracy on the size discrimination task. In fact, other studies have observed that velocity control negatively affects ability to discriminate object size as compared to position control^15,42^. It is plausible that use of a position control schema or an additional stimulation channel that could continuously convey grasp aperture information may have provided useful proprioceptive feedback to perform the size discrimination task accurately.

The object discrimination task for this study was intentionally designed to be flexible and did not constrain the subjects’ behavior to a rigid protocol. This resulted in multiple confounding factors that must be considered when attempting to explain overall task performance. Our results indicate that for both subjects, the rate of change of stimulation and peak stimulation amplitude encoded object compliance. However, only Subject 2 seemed to utilize this information to determine object compliance (up to 60% accuracy). Subject 2’s performance may be attributed to a better ability than Subject 1 in discriminating the rate of change of stimulation (using the virtual DEKA) and the peak stimulus amplitude (using the real DEKA). However, the control strategy employed in either subject may have also affected the utility of the feedback they received. When interacting with objects, Subject 1 made frequent ballistic movements of the contralateral hand that resulted in rapid changes in grasp aperture (Supplementary Video 1). Differences in stimulation dynamics across different compliances, such as the rate of change of stimulation (using the virtual DEKA) and the peak stimulus amplitude (using the real DEKA), may have been too subtle to be detected for such short durations of stimulation (or object contact). In contrast, for Subject 2, a fixed closing velocity ensured that the dynamics of stimulation were consistent for objects with the same compliance. Controlling the closing velocity of the prosthesis may have provided Subject 1 a reliable estimate of compliance and improved performance on the compliance discrimination task.

Recent studies have achieved slightly improved performance in object discrimination tasks when using biomimetic stimulation designs^15,43^. We did not employ this stimulation strategy here. However, such an approach is unlikely to overcome the limitations of the control schema used in this study.

Overall, features of stimulation encode specific physical properties of objects used during the task. However, the design of the task and control strategy of the prosthetic determine whether these features can be reliably detected by the subject. Studies that mapped grasp aperture to a tactile or proprioceptive percept have achieved the best results in discriminating both object size and compliance^18,19^. Our control schema consistently lacked one stream of information for each of our subjects.

### Considerations for closed-loop prosthesis design

In this study, we demonstrated that SCS provides somatosensory feedback that subjects can use to identify the size or compliance of objects. However, there are several shortcomings that should be addressed in future work. The percutaneous SCS system described here was implanted for up to 29 days in both subjects. Initial experiments focused on mapping evoked percepts and studying their psychophysics^25^. This information is vital to determining the electrodes and stimulation parameters to use during the discrimination tasks studied here. However, this also constrained the amount of experimental time to study these functional tasks. Neither subject had prior experience using a prosthesis, and these tasks were performed after only limited practice controlling the prosthesis in the presence of sensory feedback. It is likely that over time, subjects could learn to attend to specific changes in the stimulation and improve their performance on the object discrimination tasks. Future work should focus on tracking subject performance across longer time periods.

Previously reported psychophysics data showed that both subjects could discriminate three specific intensity levels (82% and 79% accuracy) based on trains of stimulation that had three discrete amplitudes^25^. In the present study, somatosensory feedback was modulated in real-time. Therefore, subjects had to attend to stimulation dynamics (rate of change, peak amplitude) instead of the instantaneous intensity of the evoked percept. However, our psychophysics testing did not quantify subjects’ ability to detect changes in stimulation dynamics. In fact, identifying the stimulation encoding scheme that provided the best discrimination of different objects was a major challenge. Future work should focus on characterizing the threshold and just-noticeable difference for dynamic properties of stimulation to identify the optimal stimulation encoding scheme.

Additionally, it is worth noting that the object discrimination task used in this study and several other studies^16,18,19,44^ is essentially a modified magnitude discrimination task. When all other object properties are held constant, a single stimulation feature (e.g. rate of change of stimulation) can encode a distinct property of an object (e.g. deformation). Subjects that can perceive gradation in this feature will demonstrate higher accuracy. However, these results may not generalize to a real-world somatosensory neuroprosthesis. When presented with a novel object, the same features of stimulation may encode multiple physical properties (e.g. deformation and movement of an object) so more complex stimulation schemes may be required. Most currently available prosthetic hands use very simple control schemes, such as contralateral shoulder shrug (i.e. for cable-driven devices) or low degree-of-freedom linear myoelectric control, to control grasp aperture. With these control schemes, the sensory feedback described in this study may be sufficient to provide meaningful information to a prosthetic user, although studies that monitor subject performance during activities of daily living and novel interactions are necessary to characterize the functional utility of artificial somatosensory feedback^22–24^.

## Supplementary Information

Supplementary Video S1.

Supplementary Video S2.

Supplementary Information 1.

Supplementary Information 2.

Supplementary Information 3.

## Data Availability

All data used and/or analysed during this study are included in this published article and its supplementary information files “Supplementary Data.csv” and “Supplementary Data Legend.txt”.

## References

[1] Wolf EJ (2019). Advanced technologies for intuitive control and sensation of prosthetics. Biomed. Eng. Lett..

[2] Raspopovic S, Valle G, Petrini FM (2021). Sensory feedback for limb prostheses in amputees. Nat. Mater..

[3] Committee on the Use of Selected Assistive Products and Technologies in Eliminating or Reducing the Effects of Impairments, Board on Health Care Services, Health and Medicine Division, and National Academies of Sciences, Engineering, and Medicine, *The Promise of Assistive Technology to Enhance Activity and Work Participation,* 24740 (National Academies Press, 2017). 10.17226/24740. 28910067

[4] Cordella F (2016). Literature review on needs of upper limb prosthesis users. Front. Neurosci..

[5] Gonzalez J, Soma H, Sekine M, Yu W (2012). Psycho-physiological assessment of a prosthetic hand sensory feedback system based on an auditory display: a preliminary study. J. NeuroEng. Rehabil..

[6] Pylatiuk C, Schulz S, Döderlein L (2007). Results of an Internet survey of myoelectric prosthetic hand users. Prosthet. Orthot. Int..

[7] Biddiss E, Beaton D, Chau T (2007). Consumer design priorities for upper limb prosthetics. Disabil. Rehabil. Assist. Technol..

[8] Wijk U, Carlsson I (2015). Forearm amputees’ views of prosthesis use and sensory feedback. J. Hand Ther..

[9] Rekant J, Fisher LE, Boninger ML, Gaunt RA, Collinger JL (2022). Amputee, clinician, and regulator perspectives on current and prospective upper extremity prosthetic technologies. Assist. Technol..

[10] Saal HP, Bensmaia SJ (2014). Touch is a team effort: interplay of submodalities in cutaneous sensibility. Trends Neurosci..

[11] Schiefer M, Tan D, Sidek SM, Tyler DJ (2016). Sensory feedback by peripheral nerve stimulation improves task performance in individuals with upper limb loss using a myoelectric prosthesis. J. Neural Eng..

[12] Mastinu E (2020). Neural feedback strategies to improve grasping coordination in neuromusculoskeletal prostheses. Sci. Rep..

[13] Clemente F (2019). Intraneural sensory feedback restores grip force control and motor coordination while using a prosthetic hand. J. Neural Eng..

[14] Zollo L (2019). Restoring tactile sensations via neural interfaces for real-time force-and-slippage closed-loop control of bionic hands. Sci. Robot..

[15] George JA (2019). Biomimetic sensory feedback through peripheral nerve stimulation improves dexterous use of a bionic hand. Sci. Robot..

[16] Raspopovic S (2014). Restoring natural sensory feedback in real-time bidirectional hand prostheses. Sci. Transl. Med..

[17] Horch K, Meek S, Taylor TG, Hutchinson DT (2011). Object discrimination with an artificial hand using electrical stimulation of peripheral tactile and proprioceptive pathways with intrafascicular electrodes. IEEE Trans. Neural Syst. Rehabil. Eng..

[18] D’Anna E (2019). A closed-loop hand prosthesis with simultaneous intraneural tactile and position feedback. Sci. Robot..

[19] Schiefer MA, Graczyk EL, Sidik SM, Tan DW, Tyler DJ (2018). Artificial tactile and proprioceptive feedback improves performance and confidence on object identification tasks. PLoS ONE.

[20] Oddo CM (2016). Intraneural stimulation elicits discrimination of textural features by artificial fingertip in intact and amputee humans. eLife.

[21] Mazzoni A (2020). Morphological neural computation restores discrimination of naturalistic textures in trans-radial amputees. Sci. Rep..

[22] Graczyk EL, Resnik L, Schiefer MA, Schmitt MS, Tyler DJ (2018). Home use of a neural-connected sensory prosthesis provides the functional and psychosocial experience of having a hand again. Sci. Rep..

[23] Cuberovic I, Gill A, Resnik LJ, Tyler DJ, Graczyk EL (2019). Learning of artificial sensation through long-term home use of a sensory-enabled prosthesis. Front. Neurosci..

[24] George JA, Davis TS, Brinton MR, Clark GA (2020). Intuitive neuromyoelectric control of a dexterous bionic arm using a modified Kalman filter. J. Neurosci. Methods.

[25] Chandrasekaran S (2020). Sensory restoration by epidural stimulation of the lateral spinal cord in upper-limb amputees. eLife.

[26] Resnik L, Klinger SL, Etter K (2014). The DEKA Arm: Its features, functionality, and evolution during the Veterans Affairs Study to optimize the DEKA Arm. Prosthet. Orthot. Int..

[27] Resnik L, Acluche F, Borgia M (2018). The DEKA hand: A multifunction prosthetic terminal device—patterns of grip usage at home. Prosthet. Orthot. Int..

[28] Todorov, E., Erez, T. & Tassa, Y. MuJoCo: A physics engine for model-based control. In *2012 IEEE/RSJ International Conference on Intelligent Robots and Systems*, 5026–5033 (2012). 10.1109/IROS.2012.6386109.

[29] Kumar, V., & Todorov, E. MuJoCo HAPTIX: A virtual reality system for hand manipulation. In *2015 IEEE-RAS 15th International Conference on Humanoid Robots (Humanoids)*, **2015**, pp. 657–663 (2015) 10.1109/HUMANOIDS.2015.7363441.

[30] Nanivadekar, A., Chandrasekaran, S., Gaunt, R. A., & Fisher, L. E. RNEL PerceptMapper. Zenodo, May 02, 2020. 10.5281/zenodo.3939649.

[31] Heming E, Sanden A, Kiss ZHT (2010). Designing a somatosensory neural prosthesis: Percepts evoked by different patterns of thalamic stimulation. J. Neural Eng..

[32] Flesher SN (2016). Intracortical microstimulation of human somatosensory cortex. Sci. Transl. Med..

[33] Noskin O (2008). Ipsilateral motor dysfunction from unilateral stroke: implications for the functional neuroanatomy of hemiparesis. J. Neurol. Neurosurg. Psychiatry.

[34] Smith LH, Kuiken TA, Hargrove LJ (2016). Evaluation of linear regression simultaneous myoelectric control using intramuscular EMG. IEEE Trans. Biomed. Eng..

[35] Tigra W (2018). A novel EMG interface for individuals with tetraplegia to pilot robot hand grasping. IEEE Trans. Neural Syst. Rehabil. Eng..

[36] Meeks D, Leblanc M (1988). Preliminary assessment of three new designs of prosthetic prehensors for upper limb amputees. Prosthet. Orthot. Int..

[37] van Rossum G, Drake FL, Van Rossum G (2010). The Python language reference, Release 3.0.1 [Repr].

[38] D’Anna E (2017). A somatotopic bidirectional hand prosthesis with transcutaneous electrical nerve stimulation based sensory feedback. Sci. Rep..

[39] Osborn LE (2018). Prosthesis with neuromorphic multilayered e-dermis perceives touch and pain. Sci. Robot..

[40] L. E. Osborn *et al.* Intracortical microstimulation of somatosensory cortex enables object identification through perceived sensations. In: *2021 43rd Annual International Conference of the IEEE Engineering in Medicine Biology Society (EMBC)*, 6259–6262. 10.1109/EMBC46164.2021.9630450 (2021). 10.1109/EMBC46164.2021.9630450 34892544

[41] Page DM (2021). Discriminability of multiple cutaneous and proprioceptive hand percepts evoked by intraneural stimulation with Utah slanted electrode arrays in human amputees. J. NeuroEng. Rehabil..

[42] Vargas L, Huang H, Zhu Y, Kamper D, Hu X (2022). Resembled tactile feedback for object recognition using a prosthetic hand. IEEE Robot. Autom. Lett..

[43] Valle G (2018). Biomimetic intraneural sensory feedback enhances sensation naturalness, tactile sensitivity, and manual dexterity in a bidirectional prosthesis. Neuron.

[44] Kluger DT (2019). Virtual reality provides an effective platform for functional evaluations of closed-loop neuromyoelectric control. IEEE Trans. Neural Syst. Rehabil. Eng..

