# Supplementary material for: Closed-loop stimulation of lateral cervical spinal cord in upper-limb amputees to enable sensory discrimination: a case study
Source: Sci Rep. 2022 Oct 11;12:17002. doi: 10.1038/s41598-022-21264-7 (PMC9553970; doi:10.1038/s41598-022-21264-7)
Supplement: Supplementary file 5 — Supplementary Information 3. [file 41598_2022_21264_MOESM5_ESM.pdf]

Supplementary Figures

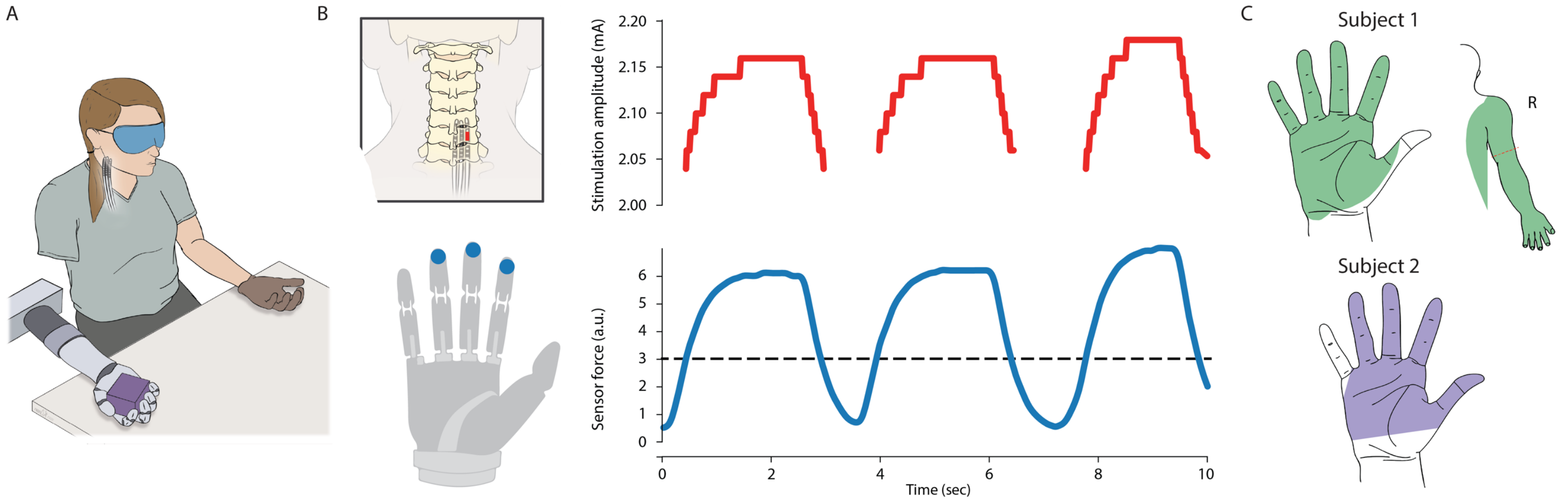

Supplementary Figure 1: A) Schematic of object discrimination task setup. B) Instantaneous sensor force (blue) and stimulation amplitude (red) during one object presentation. In this example, the subject grasped the object three times. Force sensors were located on the index, middle, and ring fingers of the DEKA hand. The maximum value across all three sensors (blue) was used to encode the stimulation amplitude. Stimulation was triggered when the sensor force crossed the threshold (dashed line). C) Representative sensory percepts for Subjects 1 and 2 generated by SCS**.** Colored areas represent the stable projected fields of the electrodes that were used during closed loop object discrimination tasks. Simultaneously evoked percepts in the residual arm are shown for Subject 1. Illustrations in (A,B) created by Kenzie Green and published under a CC BY open access license.

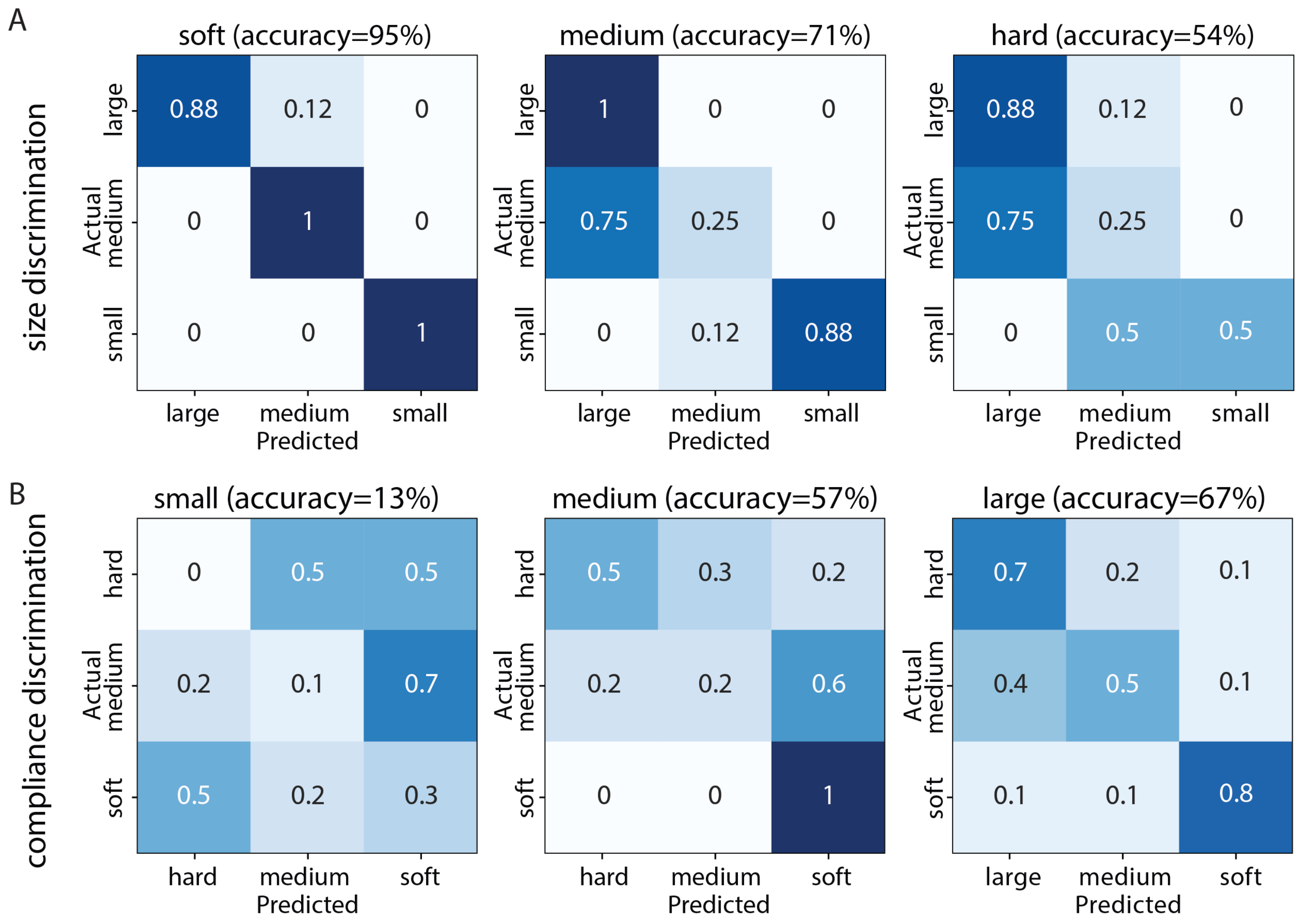

Supplementary Figure 2: Confusion matrices for the performance of Subject 1 on the A) compliance discrimination task for each object size and the B) size discrimination task for each object compliance using the virtual DEKA hand.

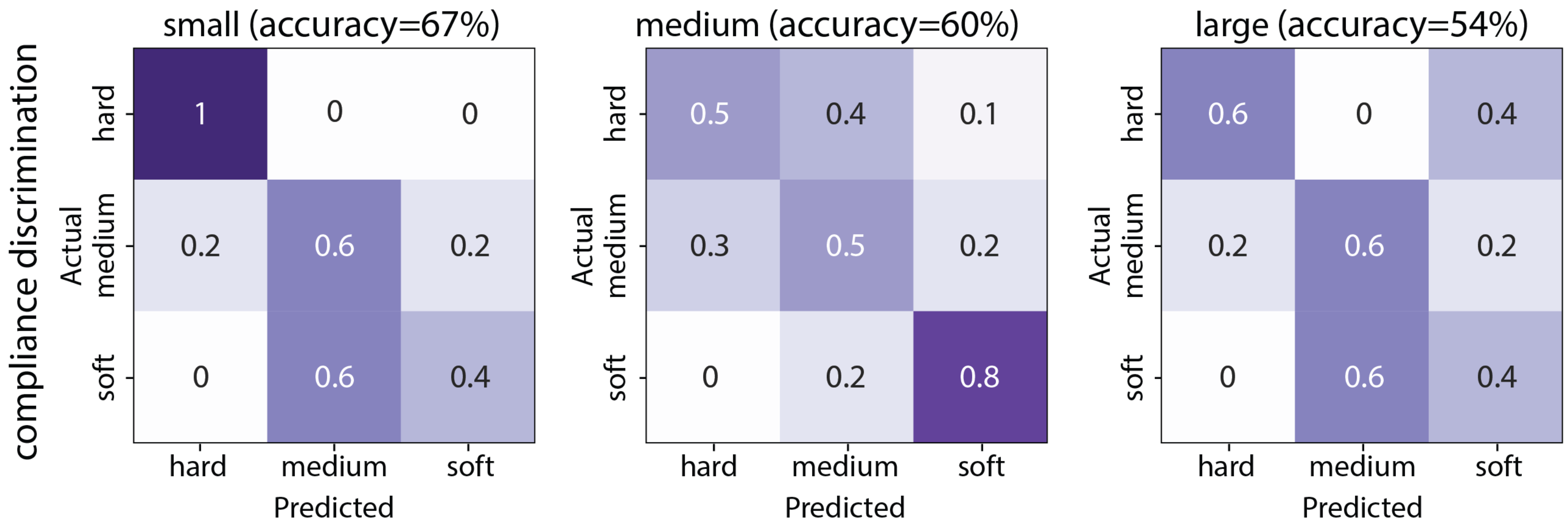

Supplementary Figure 3: Confusion matrices for the performance of Subject 2 on the compliance discrimination task for each object size using the physical DEKA hand.

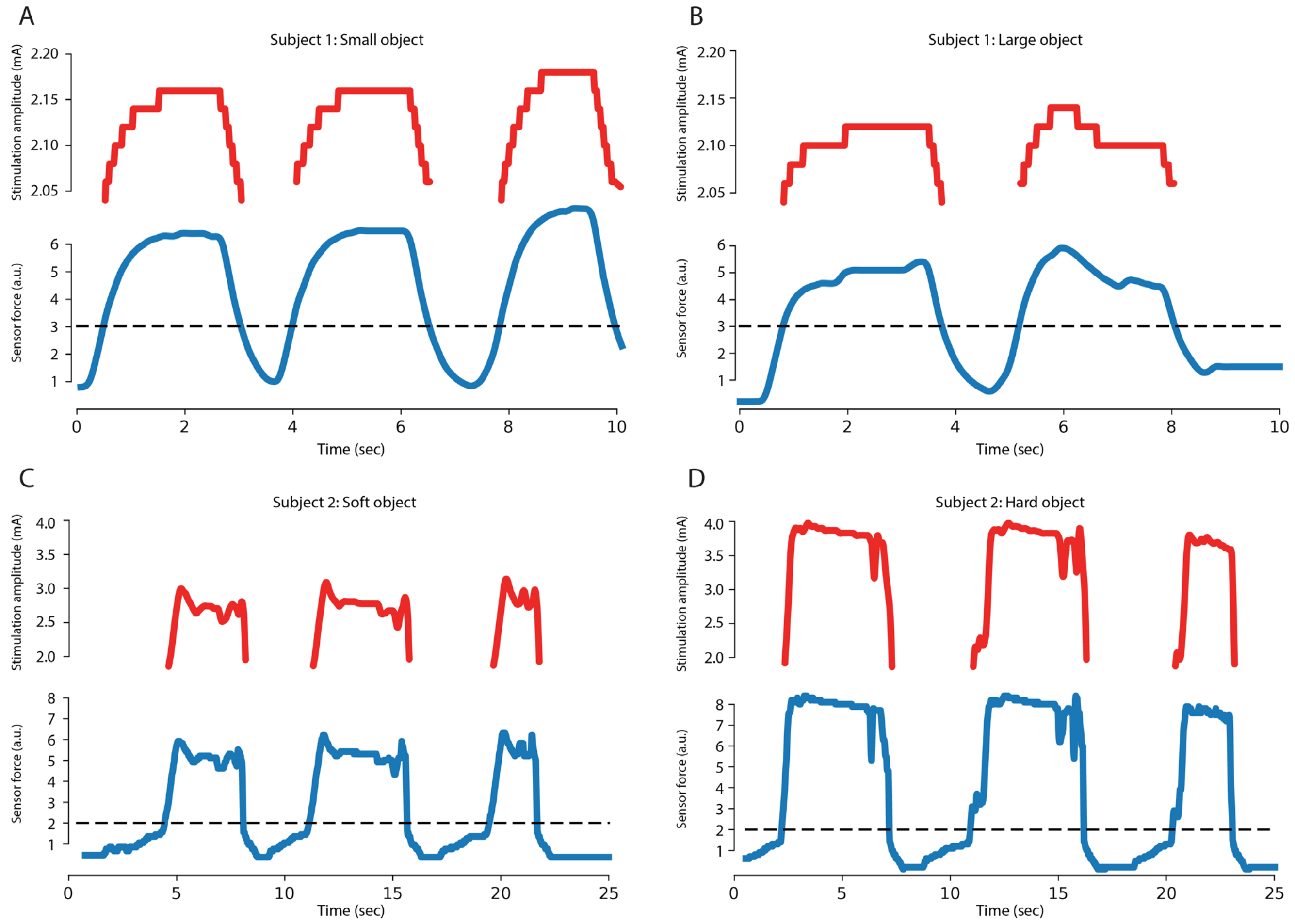

Supplementary Figure 4: Examples of sensor force (blue) and instantaneous stimulation amplitude (red) for the object discrimination task using the real DEKA hand. For Subject 1 a single trial of the size discrimination task where a A) small and B) large object were presented is shown. For Subject 2, a single trial of the compliance discrimination task where a C) soft and D) hard object were presented is shown. The deflections in the sensor force above the threshold (dashed line) triggered stimulation.

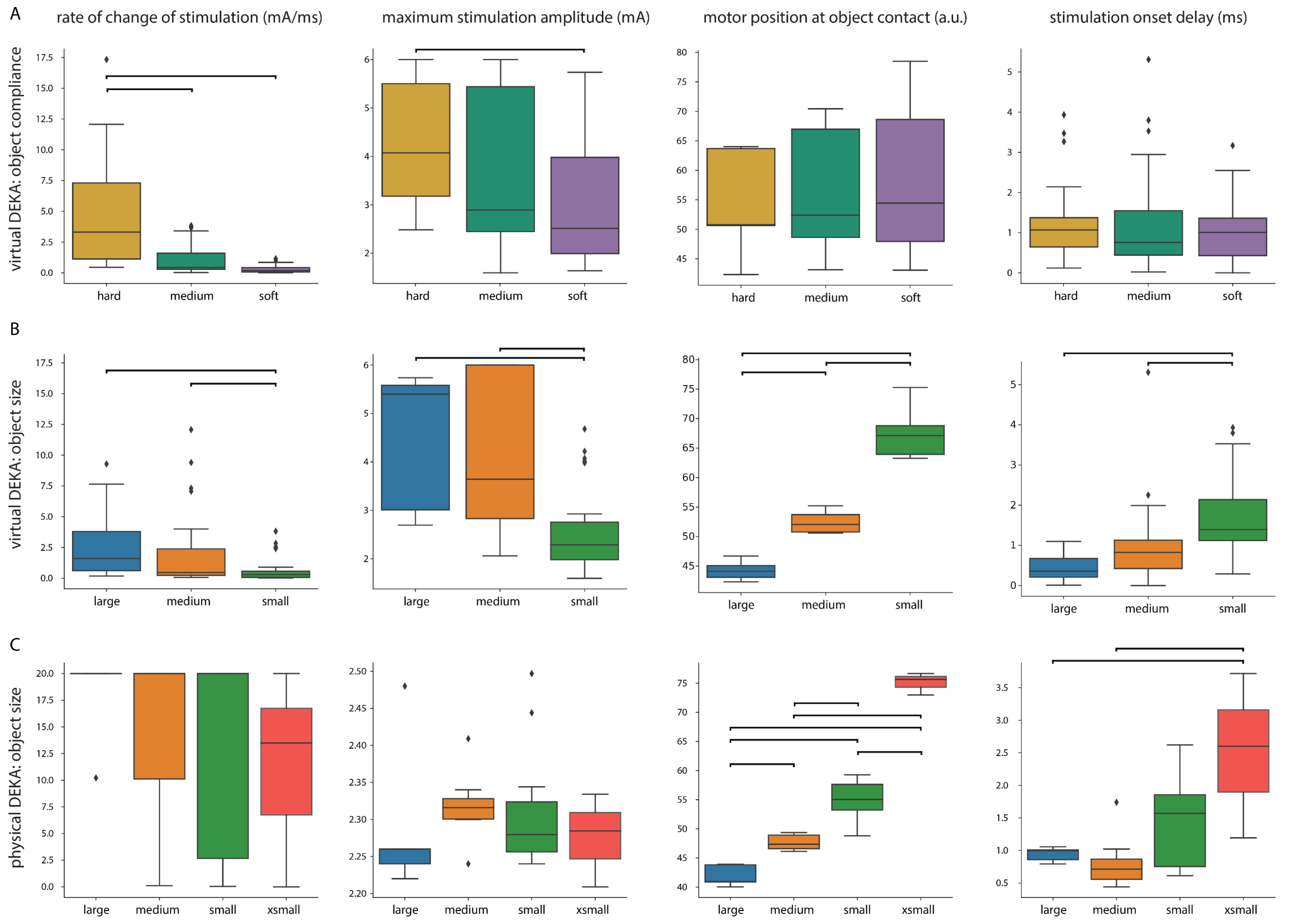

Supplementary Figure 5: Distribution of rate of change of stimulation, peak stimulation amplitude, grasp aperture at stimulation onset and stimulation onset delay using the A) virtual DEKA hand for each object compliance, B) virtual DEKA hand for each object size, C) physical DEKA hand for each object size for Subject 1. Horizontal bars denote objects that showed a significant difference ($p<0.05$, ANOVA and post-hoc comparisons) for the corresponding feature.

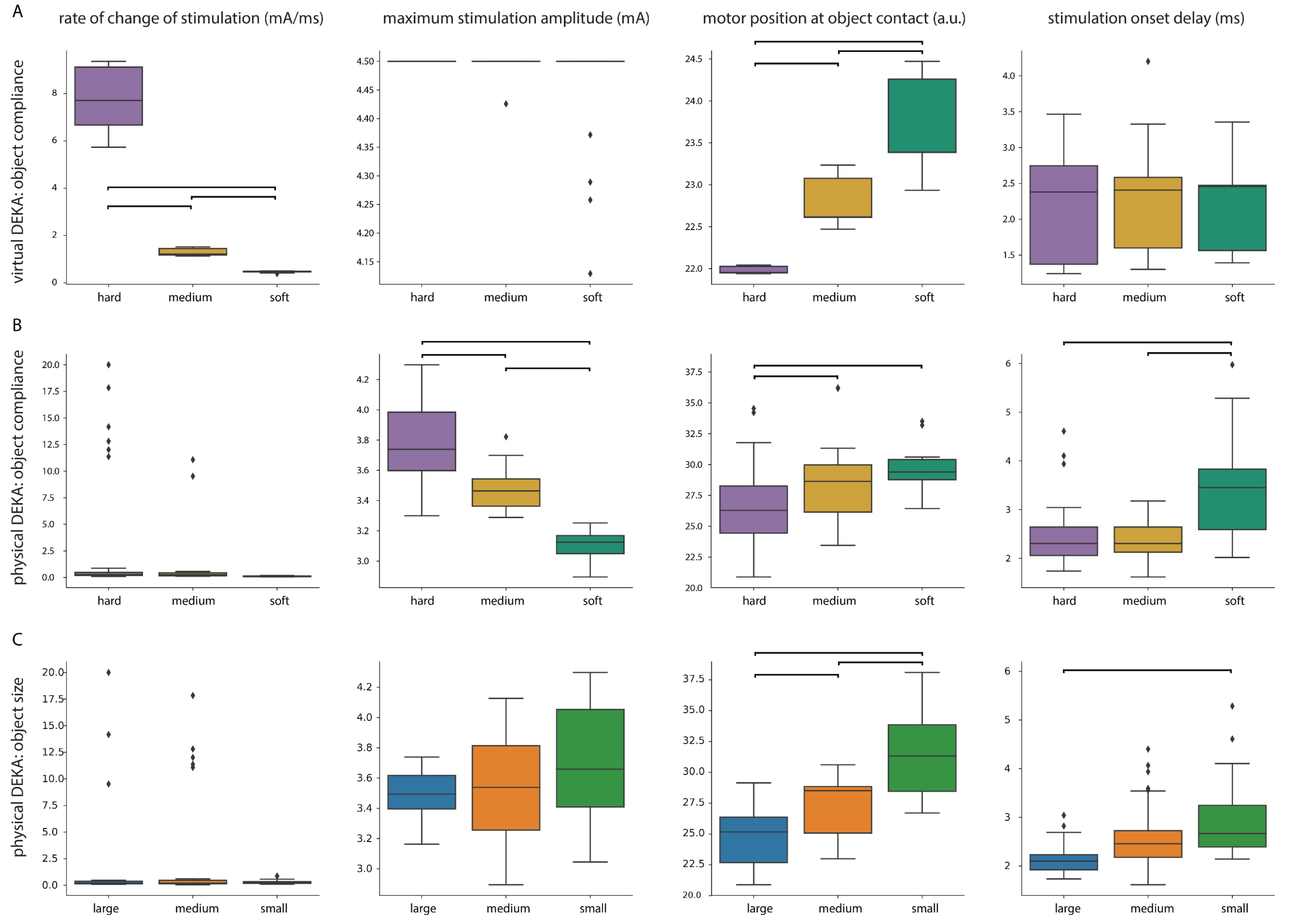

Supplementary Figure 6: Distribution of rate of change of stimulation, peak stimulation amplitude, grasp aperture at stimulation onset and stimulation onset delay using the A) virtual DEKA hand for each object compliance, B) physical DEKA hand for each object compliance, C) physical DEKA hand for each object size for Subject 2. Horizontal bars denote objects that showed a significant difference ($p<0.05$, ANOVA and post-hoc copmarisons) for the corresponding feature.
